# The impact of the suppression of highly connected protein interactions on the corona virus infection

**DOI:** 10.1038/s41598-022-13373-0

**Published:** 2022-06-02

**Authors:** Felipe Torres, Miguel Kiwi, Ivan K. Schuller

**Affiliations:** 1 Departamento de Física, Facultad de Ciencias, Universidad de Chile, Casilla 653, 78000024 Santiago, Chile; 2 Centro de Nanociencia y Nanotecnología CEDENNA, Avda. Ecuador 3493, Estación Central, 9170124 Santiago, Chile; 3 Department of Physics and Center for Advanced Nanoscience, University of California San Diego, La Jolla, CA 92093 USA

**Keywords:** Physics, Applied physics

## Abstract

Several highly effective Covid-19 vaccines are in emergency use, although more-infectious coronavirus strains, could delay the end of the pandemic even further. Because of this, it is highly desirable to develop fast antiviral drug treatments to accelerate the lasting immunity against the virus. From a theoretical perspective, computational approaches are useful tools for antiviral drug development based on the data analysis of gene expression, chemical structure, molecular pathway, and protein interaction mapping. This work studies the structural stability of virus–host interactome networks based on the graphical representation of virus–host protein interactions as vertices or nodes connected by commonly shared proteins. These graphical network visualization methods are analogous to those use in the design of artificial neural networks in neuromorphic computing. In standard protein-node-based network representation, virus–host interaction merges with virus–protein and host–protein networks, introducing redundant links associated with the internal virus and host networks. On the contrary, our approach provides a direct geometrical representation of viral infection structure and allows the effective and fast detection of the structural robustness of the virus–host network through proteins removal. This method was validated by applying it to H1N1 and HIV viruses, in which we were able to pinpoint the changes in the Interactome Network produced by known vaccines. The application of this method to the SARS-CoV-2 virus–host protein interactome implies that nonstructural proteins nsp4, nsp12, nsp16, the nuclear pore membrane glycoprotein NUP210, and ubiquitin specific peptidase USP54 play a crucial role in the viral infection, and their removal may provide an efficient therapy. This method may be extended to any new mutations or other viruses for which the Interactome Network is experimentally determined. Since time is of the essence, because of the impact of more-infectious strains on controlling the spread of the virus, this method may be a useful tool for novel antiviral therapies.

## Introduction

The current health emergency caused by the SARS-CoV-2 infection has prompted worldwide efforts to develop an antiviral treatment against Covid-19. The development of antiviral drugs requires an urgent, in-depth understanding of host–coronavirus protein–protein interactions. Many improvements in the SARS-CoV-2 interactome have been studied recently, but a disease treatment still remains elusive. Moreover, despite the genome sequences of the SARS-CoV-2 are quite similar to SARS-CoV-1 and MERS-CoV, and there are currently several vaccines in emergency use authorizations, there is no effective antiviral drug treatment yet. Current vaccines can be less effective against new variants of SARS-CoV-2 that could spread more quickly, develop more severe disease, or be capable of evading diagnostics. On the other hand, antiviral drugs can be easily administrated, possibly transported without a cold chain and at low cost. In fact, the US government invests $18.5 billion into vaccines but $8.2 billion in antiviral drugs development because it has not yet identified a highly effective drug to treat or prevent the Covid-19 infection (https://www.nytimes.com/2021/01/30/health/covid-drugs-antivirals.html).

Based on available virus–host protein Interactome Network we developed a computational approach for the fast detection of crucial protein–protein interactions. Our approach is largely inspired on the graphical representation of multi-dimensional neural networks being used in various implementations of neuromorphic computing. These maybe potential targets for disruption of the Interactome Network. When applied to the H1N1 and HIV viruses this analysis method is able to pinpoint a major part of the known proteins that are used in therapeutic treatments of this viruses^1,2^.

We have applied this method to the MERS-CoV, SARS-CoV-1 and SARS-CoV-2 to predict potential key proteins whose removal may produce important disruptions of the host–virus protein network. We demonstrate that targeting nonstructural proteins nsp4, nsp12, and nsp16 removal produces a significant structural change of the host–SARS-CoV-2 interactions. This implies that the inhibition of a small set of virus–host protein interactions profoundly impacts viral infection caused by the SARS-CoV-2 coronavirus. Furthermore, these studies also reveal that the SARS-CoV-2 infection extends across the entire human cell, unlike in the case of SARS-CoV-1 and MERS-CoV. Our approach is a useful, fast, predictive tool which helps accelerate understanding of new variant strain of SARS-CoV-2 providing new perspectives for antiviral treatment. Since COVID infections are very time-sensitive, it may be worthwhile to include this method in the therapeutic toolkit immediately, without a lengthy way for complete proof of its efficacy.

The modern computational/data science approach harnesses the symbiotic relationship between computational and biological systems, which has given rise to bioinspired or neuromorphic computing^3,4^, to developing innovative algorithms to solve complex problem. Bioinspired computation is a very promising approach including heuristic algorithms of evolution, optimization, and artificial intelligence, which allow understanding the complexity of biological systems. These methods are a very powerful predictive tool to tackle diverse problems from the analysis of uncorrelated data.

### SARS-COV-2 description

The SARS-CoV-2 coronavirus is the most recent and transmissible member of the family Coronaviridae causing the Covid-19 disease. It has a lower death rate than previous highly pathogenic viruses MERS-CoV, and SARS-CoV-1^5–8^. SARS-CoV-2 virus consist of four structural (S, N, M, E) and 16 nonstructural (nsp1–nsp16) proteins^9–12^. The spike glycoprotein (S), which lies on the particle's surface, allows to bind the cellular receptor^13–16^. While the membrane (M)^13,14,17^ is responsible for viral assembly, the envelope (E) protein is involved in release process of the genetic material^13,14,18^. Nonstructural proteins prevent the host immune response against the viral replication mediated by the nucleocapsid (N) protein^13,14,19^. Nonstructural proteins functions include inhibitory activities of the host immune response (nsp1)^20,21^; perturbation of host intracellular signaling (nsp2)^22,23^; polyprotein processing (nsp3)^24–27^, and (nsp5)^28,29^; viral replication and transcription (nsp4, nsp12)^30–33^; autophagosome (nsp6)^34,35^; RNA primase function (nsp7, nsp8, nsp9, nsp10, nsp11)^11,33,36–38^; helicase core domain that binds ATP (nsp13)^39–41^; mRNA capping (nsp14, nsp16)^11^; evasion of host cell dsRNA sensors (nsp15)^9,42^.

### Protein network

The host–virus analysis based on protein–protein interactions (PPI) network is an efficient computational approach to elucidate genetic features, molecular interactions, viral infection mechanisms, and host response^43–45^. However, to achieve this goal, a suitable amount of data is required, for instance, molecular data, genomic, and protein sequence of the virus^46^. Unfortunately, there is a paucity of SARS-CoV-2 infection mechanism data^47,48^. In this context, our model can accelerate the discovery process by elucidating the importance of new protein clusters in the infection process and identify emergent features from uncorrelated data, improving antiviral treatment development. Furthermore, our approach is a useful tool to rapidly identify potential target proteins of new variant strains of SARS-CoV-2.

Previous works focused mainly on viral protein properties within the host–virus PPI network by studying the PPI network's structural properties, local connectivity distribution, and cluster formation^43–46,48,49^. We propose an alternative approach based on the complexity and tolerance of the virus/protein–host/protein interaction network. This approach builds on the assumption that viral infection can be graphically represented by a network, where each node represents a virus–host protein interaction, and the edges correspond to proteins involved in each of those interactions. In virus–host systems, host–host protein interactions are commonly affected by viral proteins since, frequently, proteins involved in a host–host interaction also take part in a virus–host interaction^50–52^. Thus, the protein–protein connection redundancy leads to a robust virus–host network, which is unaffected by the host/protein network variations^53,54^.

On the other hand, during the viral infection, pathogen proteins mutate, changing the structural and topological properties of the network^50,55,56^. To reduce the statistical bias, we simulate the viral evolution starting from a fully connected virus–host network, where proteins interact with each other only if they are in physical contact Therefore, the schematic representation of the virus–host interaction is not affected by the virus/protein network and host/protein network structures.”

During the viral infection, pathogen and cellular proteins compete for binding partners changing their protein–protein network structure^50,51^. For instance, mutations at the protein interfaces change the “protein electrostatics and structural properties”^50^. Viral infection evolution can be represented as a protein–protein interactions network where patterns of interactions encode complex biological processes^56,57^, and statistical methods can be used for drug target identification^58^.

The efficiency of virus transmission, replication and proliferation can be identified from the effect on the connectivity of this network caused by a virus–host protein removal. The idea is that identification of the main virus–host interactions is therefore the key for the development of antiviral drugs.

In network science many systems exhibit tolerance against errors. The ability to maintain interactions or communication, notwithstanding the structural changes caused by removing nodes, arises from redundant interconnections. Many networks in nature (or real networks) manifest high tolerance against local failures, but there are still target nodes whose removal causes a significant global impact on the network's structural properties^59,60^. Inspired by this feature we explore how removal of a particular virus–host protein interaction modifies the viral infection.

### The method

We built a virus/protein–host/protein interaction network based on a public PPI database as follows, each PPI consists of one viral protein and one host protein; if two PPI share a common virus/protein or a host/protein, then they are connected. All the information of viral infection is encoded into the virus/protein–host/protein interaction network architecture. The removal of a specific protein produces a structural change in the network. We set the average connectivity (see Supplementary Information Section Fig. 9) as a proxy of the network tolerance against the deletion of proteins. We identify the target viral proteins as the proteins whose removal causes a significant variation of the network's average connectivity.

### Graph theory

**Figure 1:**
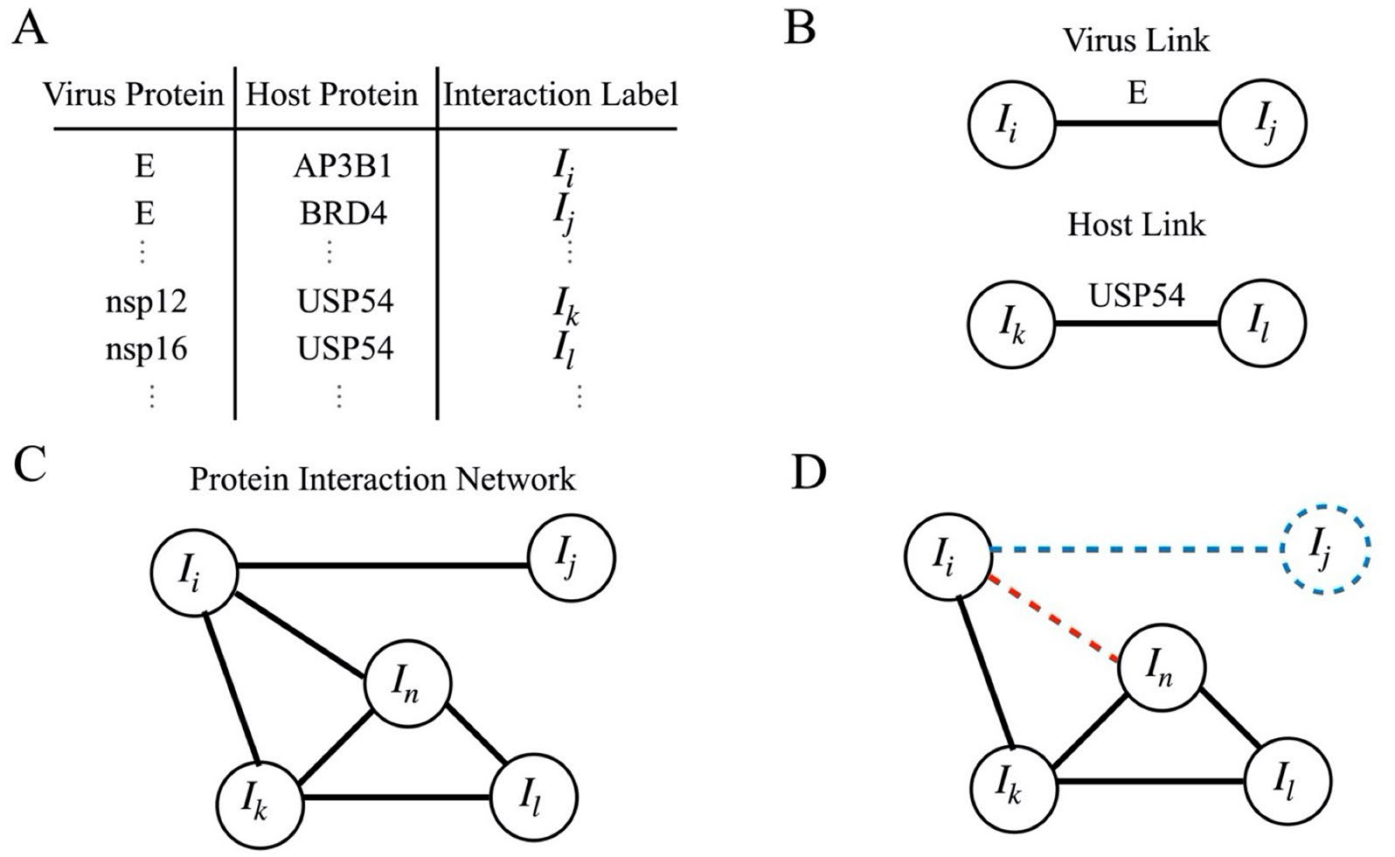
Schematic illustration of protein interaction network construction. (**A**) The protein-interaction-based networks are constructed from a public dataset reported available at http://viruses.string-db.org/ (for H1N1 and HIV) and at Krogan group’s (10.1126/science.abe9403) (for MERS-CoV, SARS-CoV-1, and SARS-CoV-2). (**B**) Each of the virus–host protein interactions is assigned an interaction label. These interactions are connected if they share a common virus protein or a host protein. (**C**) With this information, we create the protein-interaction-based adjacency matrix and network. (**D**) When a single virus protein or host protein is removed all the links that contain it disappear (red dash line), while if a single interaction is removed all its links drop out (blue dash line). We apply this process to the three viruses, MERS-CoV, SARS-CoV-1, and SARS-CoV-2, with different sets of statistical data high-score, MIST, Saint, K score.

Virus/protein–host/protein interaction network are composed by a set of interactions $I_{i} ,\;I_{j} , \ldots ,I_{k} ,\;I_{l}$, which in turn indicates the interaction between a virus protein and a host protein, as shown in Fig. 1A. Figure 1B Two interactions are connected if they share a common virus protein or a host protein, if they share both proteins then they are the same virus–host interaction. For instance, the interaction $I_{i}$, and $I_{j}$ share the E protein so they are connected (Fig. 1C). We construct a graph network with the connected interaction, and we obtain a hierarchical cluster decomposition of the interactions where each cluster is located a specific cellular compartment except the SARS-CoV-2 largest cluster which is formed by interaction with different cellular localization (Fig. 1D). When a single virus protein or host protein is removed all the links that contain it disappear, while if a single interaction is removed all its links are drop out.

### Validation

A multiply connected random network is formed by clusters which are connected by multiple alternative connections. An important way to characterize such a network is given by the average connectivity of clusters as a function of the number of nodes (or interactions in our case). For a randomly fully connected network this graph has a slope of 1 and any deviation from this reflects peculiarities and biases of the interactions and/or connectivity which is an intrinsic feature of real networks^61,62^. Hepatitis and Ebola virus network architectures are composed of a few fully connected nodes and thus our methods are not applicable.

**Figure 2:**
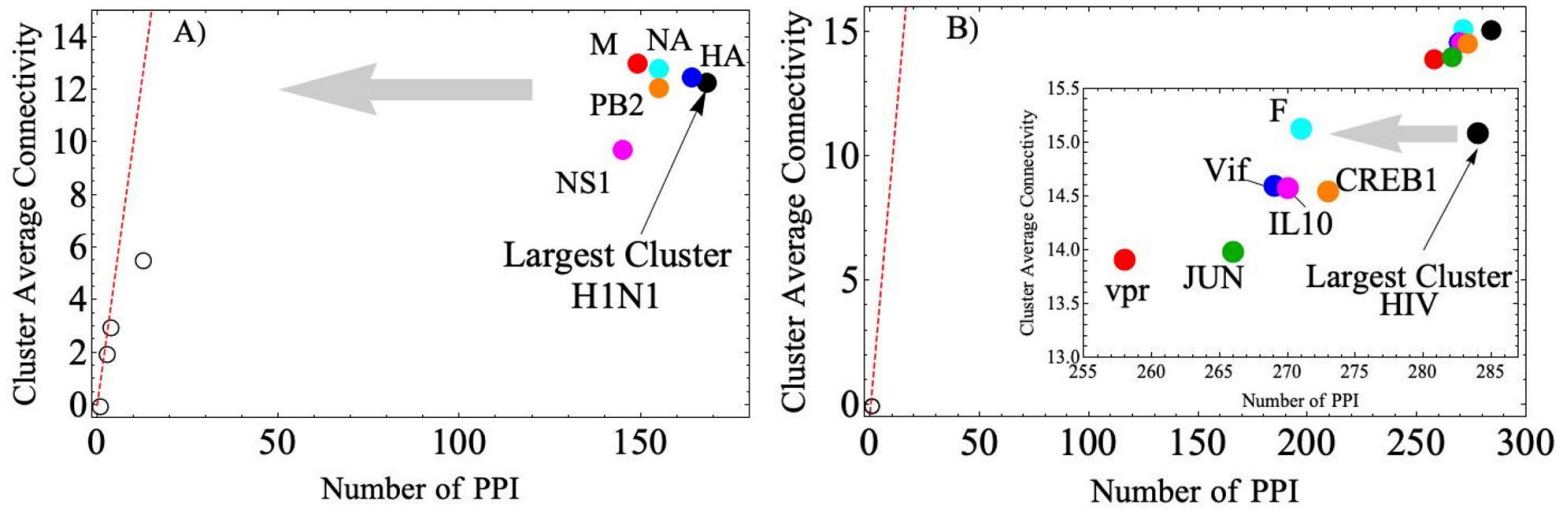
H1N1 and HIV protein interaction network. Virus/protein–host/protein interaction network for (**A**) H1N1, and (**B**) HIV. The small clusters display a linear dependence (red dashed line) between the cluster average connectivity and the number of PPI, as expected from random networks. The largest cluster (black dot) on the other hand is off this straight line, indicating a weak connectivity to the rest of the network. When certain proteins (colored dots) are removed from the network, the largest cluster moves (gray arrow) closer to the straight line as indicated by the gray arrow.

Application of this concepts to the well-known H1N1, and HIV virus (using the public PPI database http://viruses.string-db.org/ with a scoring > 0.7) reveals interesting systematics. The H1N1 (Fig. 2A) and HIV (Fig. 2B) virus/protein–host/protein interaction network (see Supplementary Information Section Fig. 2) displays few small clusters (empty circle marks) and one large cluster (black dot). Figure 2 shows the average connectivity of each cluster as a function of the number of PPI. The dashed line is a line with slope of 1 expected from a fully randomly interconnected network^61,62^. The small clusters are on this line, however the largest cluster (black dot, for each virus) are completely off the line. This indicates that the large cluster is weakly connected to the rest of the network and therefore elimination of a few connections may produce a major disruption of network connectivity.

In Fig. 2 we show the effect of the removal of specific proteins on the position of the largest cluster on this graph. This is a measure of the tolerance of the network against PPI removal by means of the average connectivity variation of the largest cluster. Figure 2 shows that removal of some proteins from the network moves the position of the largest cluster closer to the expected random network curve. Interestingly all the predicted target proteins (i.e., removed proteins producing a significant change in the largest cluster connectivity) for the H1N1 virus are already used in vaccines: HA (blue) attenuated vaccine strains, NA (cyan) conserved epitopes, M (red) ectodomain based vaccines, PB2 (orange) and NS1 (magenta) live attenuated vaccine strains^1,63,64^. Figure 2B shows the same for the HIV virus. Figure 2B displays the following target proteins: F (cyan); vif (blue); IL10 (magenta); CREB1 (orange); JUN (green); vpr (red). Inactivated antigen approach to influenza vaccination promotes an immune response against the viral surface glycoproteins HA and neuraminidase NA^1,63,64^, while live attenuated influenza vaccine targets M, PB2, and NS1^63,65–68^. Our method predicted those proteins as key targets which disrupt the influenza network structure.

Our method identifies three key proteins that play a significant role in the efficacy of the vaccination against HIV-1, Vpr, vif, and CREB1. These play a crucial role in the development of therapeutic interventions as described in references^2,69,70^. Our method does not identify other protein vaccine candidates, such as multiple gp120 envelope proteins^71^, because the statistical weight associated with protein removal relies on the number of connections and the effective long-range correlation between virus–host interactions.

Our findings indicate that a study of the viral/protein–human/protein interaction in the H1N1 and HIV is able to identify important proteins used in vaccines. Considering that time is of essence, this calls for application of this method to other important viral systems although further studies will be needed as experimental data becomes available.

Geometrical descriptors of structural changes of the network like the largest cluster average connectivity and the number of PPI capture the impact of highly connected nodes. This statistical description is sensitive to the number of disconnected links, which accounts for effective long-range interactions. Therefore, we are not able to account for targets which depend on long range biological interactions. However, by evaluating different scoring methods and using many protein interactions, we decrease the statistical bias and discrepancies between predicted proteins based on biological mechanisms and statistical methods.

### Host–coronavirus protein interaction network

**Figure 3:**
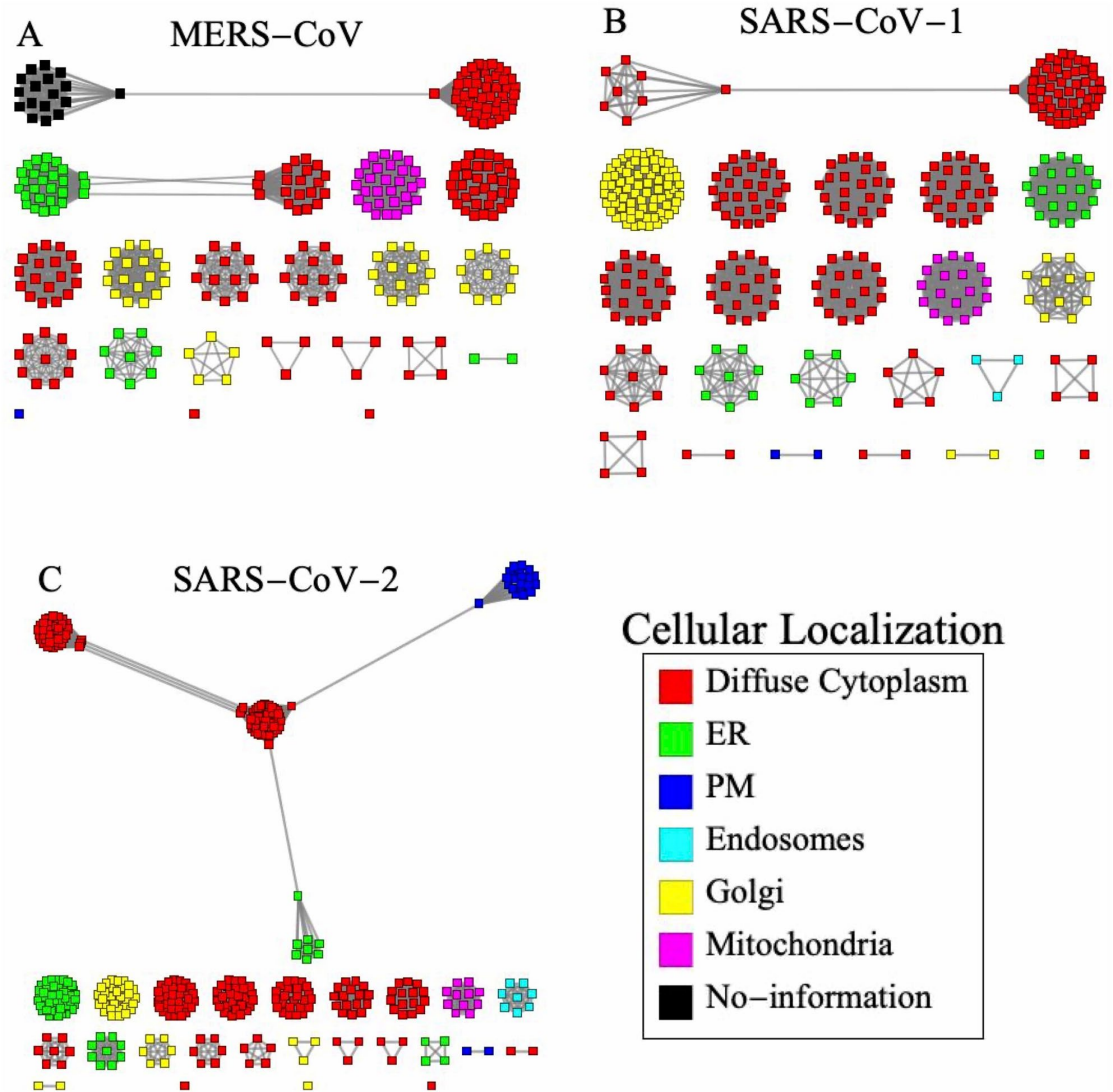
Host–coronavirus protein interaction network. High confidence scoring threshold for virus/protein–host/protein interaction network for (**A**) MERS-CoV, (**B**) SARS-CoV-1, and (**C**) SARS-CoV-2. If two virus/protein–host/protein interactions (square marks) share a common virus protein or a host protein, then they are connected by a link (black line). The hierarchical cluster structure relates to different intracellular compartments. SARS-CoV-2 largest cluster is formed by four subcluster with different cellular locations. Color-code scheme: diffuse cytoplasm (red); endoplasmic reticulum (ER) (green), plasma membrane (PM); (blue); endosomes (cyan); Golgi (yellow); mitochondria (magenta), no information (black).

**Figure 4:**
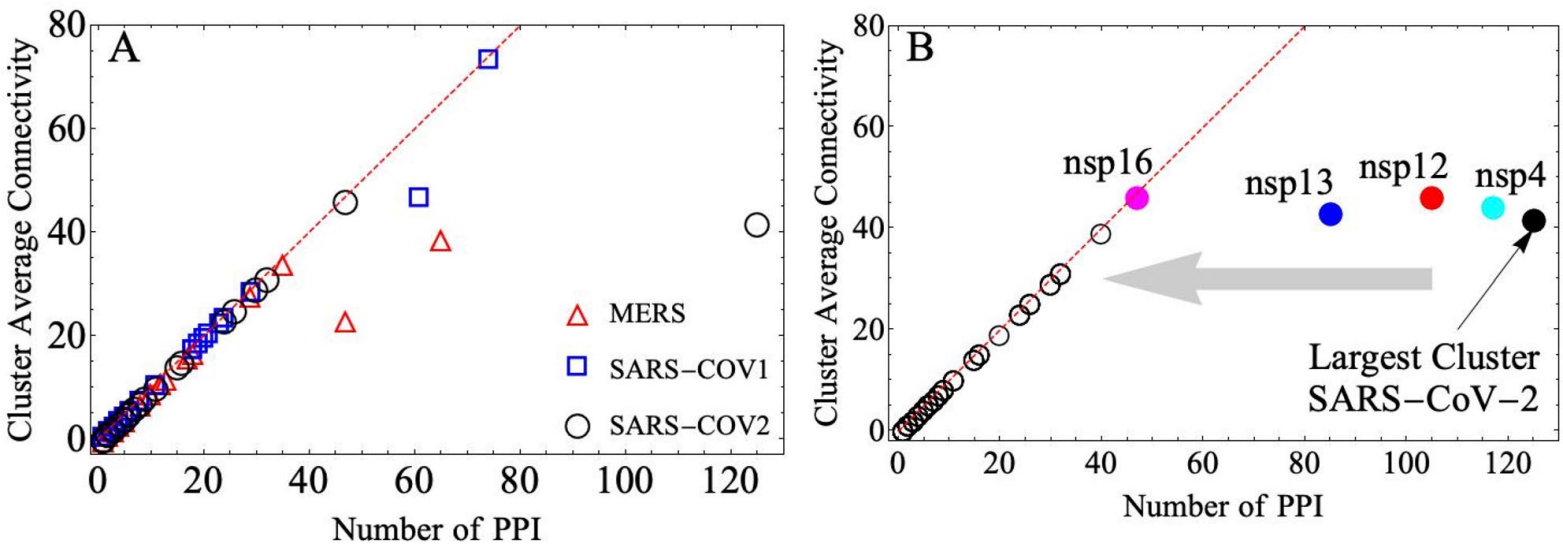
Coronavirus–host tolerance network analysis. (**A**) Cluster average connectivity dependence with the number of PPI for human respiratory syndromes associated with coronavirus (Cov). MERS-CoV (red triangle), SARS-CoV-1 (blue square), and SARS-CoV-2 (black circle). (**B**) Effect of virus protein removal on SARS-CoV-2 network connectivity. The black dot outside of this linear curve correspond to the largest coronavirus cluster. Removal of the nonstructural proteins, nsp4 (cyan dot), nsp12 (red dot), nsp13 (blue dot), or nsp16 (magenta dot), displaces substantially the largest cluster closer to the linear dependence, while the small clusters move slightly along the dashed line. The gray arrow indicates the displacement of the largest cluster position.

**Figure 5:**
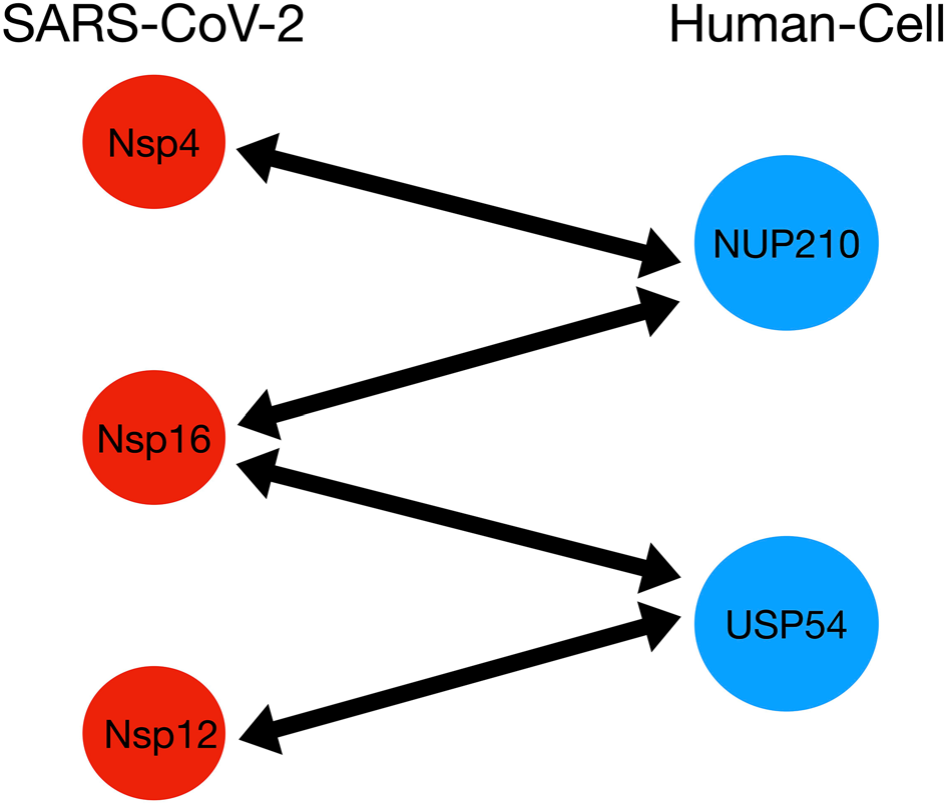
SARS-CoV-2 target protein interaction. Most disruptive set of virus–host protein interaction for SARS-CoV-2 infection. The removal of these virus–host interactions causes a significant change in the virus–host network's properties. These virus proteins belong to different cellular complexes. Nsp4 and nsp16 are localized in the same structure, whereas nsp12 appears to localize to the plasma membrane (SARS-CoV-2), diffuse cytoplasm (SARS-CoV-1), and endoplasmic reticulum (MERS-CoV). The three dimensional structures of those proteins can be extracted from https://zhanglab.ccmb.med.umich.edu/COVID-19/ (SARS-CoV-2 non-structural proteins) and https://swissmodel.expasy.org/repository/uniprot/Q70EL1 (human cell proteins).

Recently, a map of virus–host protein for MERS-CoV, SARS-CoV-1, and SARS-CoV-2 has been published^44,45^, including a high-score update and additional statistical updates of mass spectrometry and PPI scoring information^44,45^, reporting more than 300 high-confidence interactions for these coronaviruses. A protein-interaction-based network analysis based on this data set has not yet been carried out, to the best of our knowledge. Moreover, the networks tolerance against antiviral attack has not been reported. Within this context, we study the tolerance of MERS-CoV, SARS-CoV-1, and SARS-CoV-2 networks to removal of a single protein [see Supplementary Information (SI), Figs. 3–5]. We construct a protein-interaction-based network for each of these coronaviruses by identifying virus–host interactions as nodes interacting with each other by sharing a virus or a host protein (see Fig. 3). According to the information provided by Krogan’s lab group^44,45^, we use a high-confidence interactome to create host–coronaviruses networks for each of these viruses (it is important to emphasize that our analysis focuses on the architecture of the Interaction Network instead of the Proteins Network). Figure 3 shows that all networks display a hierarchical cluster structure; MERS-CoV and SARS-CoV-1 share a similar structure characterized by a largest cluster formed by two sub-clusters see Fig. 3. In contrast, the SARS-CoV-2 largest cluster is formed by four sub-clusters, which suggests that the evolution from MERS-CoV to SARS-CoV-2 is closely linked to the largest cluster topology.

By using the cellular localization analysis provided by Krogan’s lab group^44,45^, we apply a color-code [diffuse cytoplasm (red); endoplasmic reticulum (ER) (green), plasma membrane (PM); (blue); endosomes (cyan); Golgi (yellow); mitochondria (magenta); no information (black)] for the viral/protein localization, as shown in Fig. 3. In the MERS-CoV and SARS-CoV-1 network, the largest cluster appears mainly located in the diffuse cytoplasm. Meanwhile in SARS-CoV-2, the largest cluster spreads across the diffuse cytoplasm, endoplasmic reticulum, and plasma membrane. Therefore, our study reveals that the hallmark of the SARS-CoV-2 infection is a highly connected virus/protein–host/protein interaction network across the entire host cell, which may be the key to its efficient infection mechanism. These results indicate that the protein-interaction-based network exhibits a hierarchical cluster structure which is highly correlated with cellular localization. Viral/proteins interact with host/proteins while in direct physical contact. However, we find that virus–host interactions which apparently are biologically disconnected maybe linked indirectly throughout the virus/protein–host/protein interaction network. This maybe a very important feature of the SARS-CoV-2 hijacking of host cell regulation.

The virus/protein–host/protein interaction graph network is built on virus–host interactions mediating cellular functions and viral infection, which are connected if they share a common single virus/protein or host/protein. Figure 3 shows the MERS-CoV, SARS-CoV-1, and SARS-CoV-2 virus/protein–host/protein interaction network, where virus/protein–host/protein interactions are represented as square marks and the links between them indicate that two interactions share a common single virus/protein or host/protein. Affinity purification mass spectrometry and structural properties of protein–protein complexes allow the identification of these protein interactions. However, only a few of these interactions play a relevant biological role^72–74^. We compare several statistical methods to score the PPI and to create the virus/protein–host/protein interaction network (see Supplementary Information Figs. 7, 8).

To compare the infection mechanism of MERS-CoV, SARS-CoV-1, and SARS-CoV-2 we construct their virus/protein–host/protein interaction network. To quantify the effect of virus/proteins on the virus/protein–host/protein interaction, we investigate the variation of average connectivity by removing some of these elements. We use the change of the structural properties of the network as a proxy to simulate potential damage produced by antiviral activity.

### Coronavirus–host protein network analysis

Figure 4A shows that the highly pathogenic coronaviruses MERS-CoV, SARS-CoV-1, and SARS-CoV-2 display an almost linear relationship between the number of PPI and the average connectivity per cluster. Since the average connectivity of these small clusters increases with the number of PPI there is a homogenous connectivity distribution rather highly connected virus–host interactions or hubs. However, the largest clusters display key virus–host interactions, regardless of the small number of connections they have, that when they are removed several virus–host subcluster are disconnected. For this reason, the largest cluster plays a more central role in the structure of the virus–host interaction network. The largest clusters have around 40–60 PPI for MERS-CoV and SARS-CoV-1, however, SARS-CoV-2 has twice as many, see Fig. 4A.

Due to the urgency of the ongoing worldwide health emergency caused by the Covid-19, we focus on the tolerance against protein removal of SARS-CoV-2; similar analysis for the MERS-CoV, and SARS-CoV-1 are addressed in the Supplementary Information (SI) Section, Figs. 3–5. Figure 3B shows that removal of most virus proteins does not affect the linear dependence of small clusters. However, removal of nsp4, nsp12, nsp13, and nsp16 proteins, reduce substantially the number of PPIs bringing the largest cluster closer to the linear dependence. The disruptive effect of antiviral drugs affects mostly the host/protein–virus/protein interaction rather than a single viral protein. Therefore, a more useful approach is to investigate the virus–host network tolerance against single virus–host interaction removal.

### SARS-CoV-2 target protein interaction

The SARS-CoV-2 network retains its structural properties against protein removal except when nsp4, nsp12, nsp13, nsp16, are removed (see Fig. 4B and Supplementary Information (SI) section, Figs. 3–5). The comparison between MERS-CoV, SARS-CoV-1, and SARS-CoV-2 implies that common proteins are located in similar places and thus expected to perform the same functions. The exception is nsp13, which change its location from virus to virus thus expected to exhibit different functions and therefore is not included in our analysis^44,45^. Moreover, we found that the set of virus–host interactions causing a significant change in the virus–host network appears in a small cluster linking the nsp4, nsp12, and nsp16 proteins, which belong to different complexes (Fig. 5). This provides a target which produces significant changes in the virus–host network and thus is the optimal candidate for an antiviral drug attack.

### Comparative scoring methods analysis

We use four protein–protein interaction scoring methods, high confidence, MIST, Saint, and K, as published on^44,45^. All these scoring methods reveal the importance of the SARS-CoV-2 largest cluster on the virus–human interaction. MIST and Saint scoring methods are based on experimental biological data ranking the confidence of the virus/protein–host–protein interactions between 0 and 1, where 1 indicates the maximum confidence value (we selected PPI with a confidence ≥ of 0.6). Combining the MIST and Saint scoring method with protein complexes information, Krogan’s lab group introduce a high confidence score^44,45^. Finally, the K score is defined as the average between the MIST and Saint score^44,45^. In this case, we also use a confidence score ≥ 0.6. We identify critical targets by removing a PPI that produce a significant variation on the SARS-CoV-2 largest cluster connectivity. As shown in Table 1, all the scoring methods identify the nsp4/NUP210 and nsp16/NUP210 interactions as critical targets. The nsp12/USP54 interaction is detected only by the High confidence scoring method since the nsp12 protein is not included in the MIST, Saint, and K dataset. This comparative analysis reveals that our method allows us to identify crucial PPI in good agreement with different scoring methods; thus, our approach is a robust predictive method (see Table 1).

**Table 1:** Comparative analysis of the different scoring methods.

PPI	High Confidence	MIST	Saint	K
**nsp4/NUP210**	**X**	**X**	**X**	**X**
nsp4/ALG11			**X**	
nsp7/ALG11			**X**	
**nsp12/USP54**	**X**	No data	No data	No data
**nsp16/NUP210**	**X**	**X**	**X**	**X**
**nsp16/USP54**	**X**			
nsp16/INTS7		**X**	**X**	**X**

Comparative analysis of high confidence, MIST, Saint, and K scoring method. We identify the PPI that produce a significant variation of the SARS-CoV-2 largest cluster connectivity. Empty boxes indicate non identified PPI.

The nsp4/NUP210 and nsp16/NUP210 interactions emerge as common key targets in all scoring methods. The nsp12/USP54 interaction appears as a key target only when we use the high confidence scoring since this protein is not included in MIST, Saint, K data set. This analysis reveals that our method allows us to detect key PPI independently of the statistical scoring method. Significant texts are in bold.

## Discussion

We developed a new, fast detection method of the key controlling virus/protein–host/protein interactions which identifies the SARS-CoV-2 infection mechanism. An analysis of the protein-interaction-based network provides a universal hierarchical cluster decomposition from three coronaviruses-host networks. This cluster decomposition identifies all virus–host interactions sharing a virus and host proteins and localizes them in different cellular compartments. We find that the SARS-CoV-2 largest cluster structure extends across the entire host cell, revealing an enhanced hijacking of the host cell regulation mechanism. The evolution of the largest clusters coincides with new coronavirus strains' suggesting that further mutational changes will occur within this structure. We verified the validity of our method by applying it to H1N1 and HIV viruses, in both cases we detect important target proteins which are used in antiviral drug development.

We simulate an inhibitory antiviral action on SARS-CoV-2 by removing a protein and virus/protein–host/protein interaction. This process shows that removal of the nonstructural protein nsp4, nsp12, nsp13, nsp16, produces a significant change in the structural properties of the virus–host network resembling the behavior of previous coronaviruses, MERS-CoV, and SARS-CoV-1. Furthermore, we find that this effect is supported exclusively by a small set of virus–host interactions linking nsp4, nsp12, and nsp16 proteins. Moreover, our protein-interaction-based network method does not depend on the scoring method for protein–protein interactions. Interestingly our cluster decomposition coincides with the previously reported clustering obtained from genetic analysis, of these three coronaviruses.

The SARS-CoV-2 infection spreads a viral interaction network across different intracellular compartments, which strongly suggests that this network's largest cluster encodes relevant mechanisms of viral infection. Our analysis highlights the significance of long-range proteins interactions resulting from emerging and collective behavior of the virus–host interactome, which may be inspired by the seeking of a new biological mechanism for viral infection. In a global health emergency, it is crucial to develop a fast viral mechanism characterization method. Our analysis based on the geometric properties and tolerance of a virus/protein–host/protein interaction network is a valuable tool for understanding the viral mechanism of new variant strains of SARS-CoV-2 and detecting target protein–protein interaction rapidly. Our insights may provide essential information for further antiviral drug development, uncover the role of nonstructural viral/proteins, and identify the importance of a small set of virus–host interactions. Furthermore, structural information of the virus/protein–host/protein interaction networks may help understand the spike protein function in the mutations of the SARS-CoV-2.

The conventional methods based on network analysis, map the virus–host interaction into a graph between the virus and human proteins where connections involve biological interactions. On the other hand, we use an interaction-based network to explore its structure and the impact caused by removing certain proteins. It is our hypothesis that this simulates the antiviral action and brings a new perspective regarding viral infection. We validate this hypothesis by applying it to the known effects of protein removal on the H1N1 and HIV viruses. This methodology applied to the SARS-CoV-2 infection, predict that removal NUP210 and USP54 host proteins, produce a major change in the connectivity of the SARS-CoV-2 interaction network. These are known, to play an important role in cancer therapy and autoimmune diseases^75,76^. Although, nonstructural proteins (nsp4, nsp12, nsp16) have not attracted attention as target proteins, our validated method predicts them to be important candidates for antiviral drug and vaccine development. According to available connectivity data the SARS Cov2 spike proteins are weekly connected to the whole network. Because of this removing them as proposed by our method, does not affect in a major way the network structure and connectivity. On the other hand, in the case of H1N1 where more connectivity data is available, we properly predict which proteins are targets^77^. The spike proteins, the glycoproteins HA, and neuraminidase proteins NA are located on the viral surface, and they are also the most variable viral proteins. Protein variability results in highly connected nodes because SARS-CoV-2 variants and mutations potentially change viral properties. The identification of spike protein is restricted by available information in this work. Notwithstanding the preceding, our method has been able to identify crucial target proteins for H1N1 and HIV viruses and revealed the role of non-structural proteins, as has been reported by other authors^62–68^.

Predicted targets proteins are validated by Live attenuated and Inactivated antigen vaccines methods. Similarly, standard antiviral therapy mainly uses inhibitors to prevent the spike and nucleocapsid proteins binding^78,79^. However, it has been reported that the non-structural proteins play a significant role in the virulence of the SARS-CoV-2 virus^80^ as our method predicts.

Molecular docking study of ivermectin, and remdesivir indicates spike, M, N, nsp14, and nsp16 as viable targets for drug treatment development of SARS-CoV-2^81,82^, while Raltegravir and Maraviroc are potential candidates to inhibit nsp-16 protein^83^. Multi-targeting features of Diosmin display highest binding affinity and inhibitory action of several non-structural proteins (nsp3, nsp9, nsp12, nsp15)^84^. This confirmation of the role of non-structural proteins in treatment validates our target proteins predictions.

Finally, our approach of graphically visualizing the connectivity of interactome networks overlaps with the design and optimization of neural networks, and as such our method could pave a roadmap to combine neuromorphic computing and artificial intelligence to optimize the design of drug treatments of viral diseases.

## Materials and methods

### Dataset

We obtain the protein sequence similarity, high-confidence protein interaction, and localization of the virus protein from a public dataset and permission as reported by Krogan’s lab group^44,45^ (10.1126/science.abe9403).

### Code

All code used in the calculations of the network analysis is available from FT upon request. Supplementary Information contains all the raw data of the simulations.

### Graph theory methods

A virus/protein–host/protein interaction network $G(I,\;k)$ is a set of $I_{1} ,\;I_{2} , \ldots ,\;I_{D}$ virus–host interactions with $k_{1} ,\;k_{2} , \ldots ,\;k_{D}$ connections between them, as described in Fig. 1. Graph representation of this network consists of D-nodes where each node corresponds to a one virus–host interaction. If two interactions share a common virus/protein or a host/protein, then they are connected by a link. The number of connections of the i-th virus–host interaction $I_{j}$ is $k_{j}$. The graphs were constructed from binary matrices where entries correspond to all virus/protein–host/protein interactions. Undirected links indicate connected virus–host protein interactions. The adjacency matrix $A_{ij}$, with $i,\;j = 1,\;2, \ldots ,\;D$, encodes all these interactions, with $A_{ij} = 1$ indicating that the i-th and j-th interaction are connected, and $A_{ij} = 0$ otherwise, Fig. 1. Notice that the connection of the i-th interaction $k_{i} = \sum\nolimits_{j = 1}^{D} {A_{ij} }$.

According to cluster decomposition, the adjacency matrix of full connected interactions can be expressed as sum of N cluster adjacency matrices

1 $$A_{ij} = \mathop \sum \limits_{L = 1}^{N} a\left( L \right)_{ij}$$

where $a(L)_{ij}$ stands the adjacency matrix of the L-th cluster. To quantify the network tolerance against local failures due to the removal of a virus protein or single virus–host interaction, we introduce the average connectivity as a proxy. This way, the cluster average connectivity is

2 $$k(L) = \frac{1}{{n_{L} }}\mathop \sum \limits_{i,j = 1}^{{n_{L} }} a(L)_{ij}$$

Here $n_{L}$ is the number of PPIs of the L-th cluster. Notice that the removal of a virus protein implies that all its interactions are deleted. In this case we have a network composed by D-1 interactions with D-1 connections.

### Network’s tolerance against the antiviral activity

We simulate the inhibitory action of antiviral drugs utilizing node removal. The features of the cluster decomposition, in particular, the properties of its larger cluster, change as virus proteins or virus–host interactions are deleted. We use the cluster average connectivity variation as a proxy to quantify the impact of virus proteins in the virus–host interaction networks, as detailed in the Supplementary Information (SI), Figs. 3–8.

## Supplementary Information

Supplementary Figures.
