# Supplementary material for: The impact of the suppression of highly connected protein interactions on the corona virus infection
Source: Sci Rep. 2022 Jun 2;12:9188. doi: 10.1038/s41598-022-13373-0 (PMC9160517; doi:10.1038/s41598-022-13373-0)
Supplement: Supplementary file 1 — Supplementary Figures. [file 41598_2022_13373_MOESM1_ESM.docx]

**Supplementary Information**

**The impact of the suppression of highly connected protein interactions on the Corona virus infection**

Felipe Torres^1,2^, Miguel Kiwi^1,2^, Ivan K. Schuller^3^.

^1^ Departamento de Física, Facultad de Ciencias, Universidad de Chile, Casilla 653, Santiago, Chile 78000024*.*

^2^ Centro para el Desarrollo de la Nanociencia y la Nanotecnología, CEDENNA, Avda. Ecuador 3493, Santiago, Chile, 9170124.

^3^ Department of Physics and Center for Advanced Nanoscience, University of California San Diego, La Jolla, California 92093, USA.


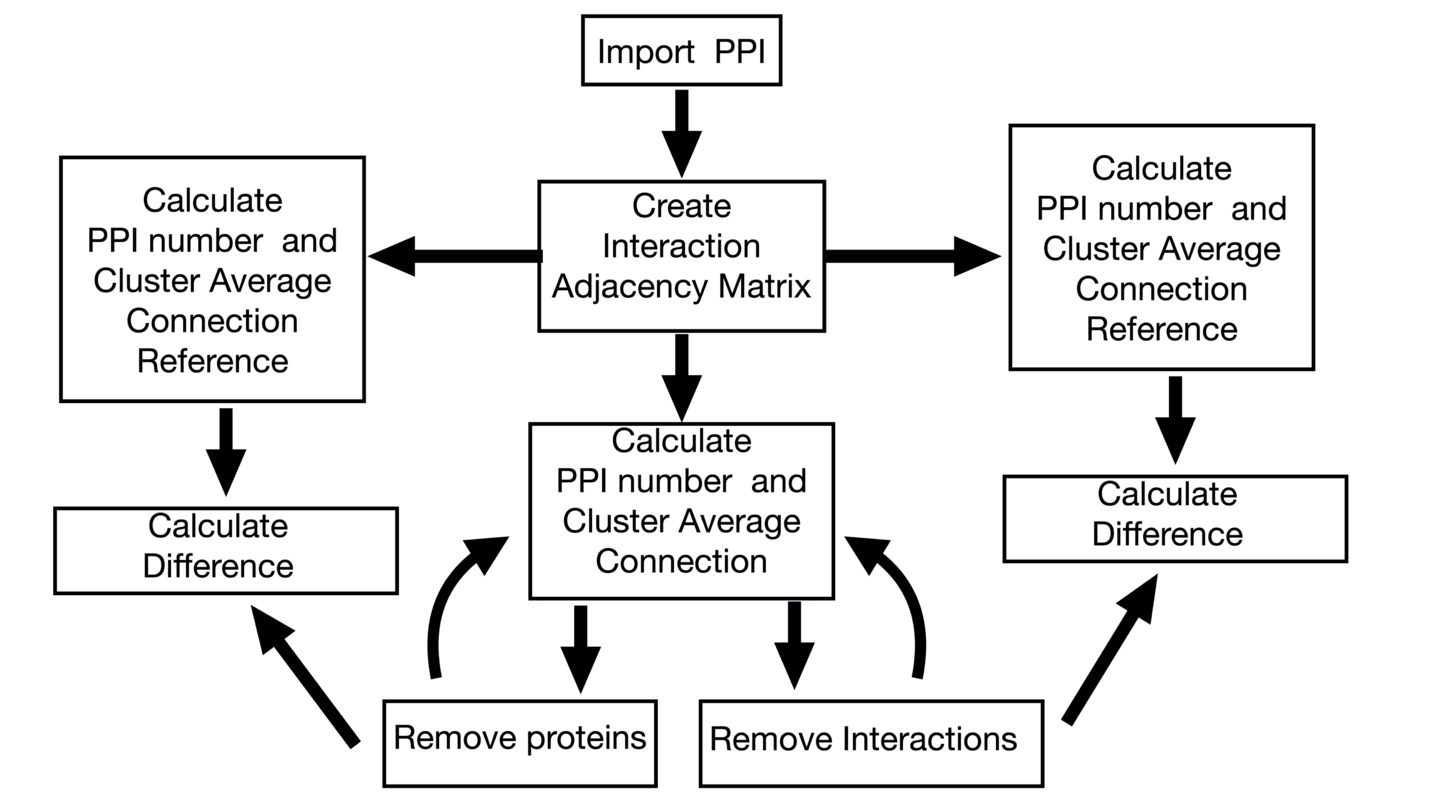


**Figure 1. Computational algorithm structure**. Schematic structure of the algorithm used to calculate the Cluster average connectivity dependence with the number of PPI. All code used in the calculations of the network analysis is available from FT upon request. Supplementary information contains all the raw data of the simulations.

| 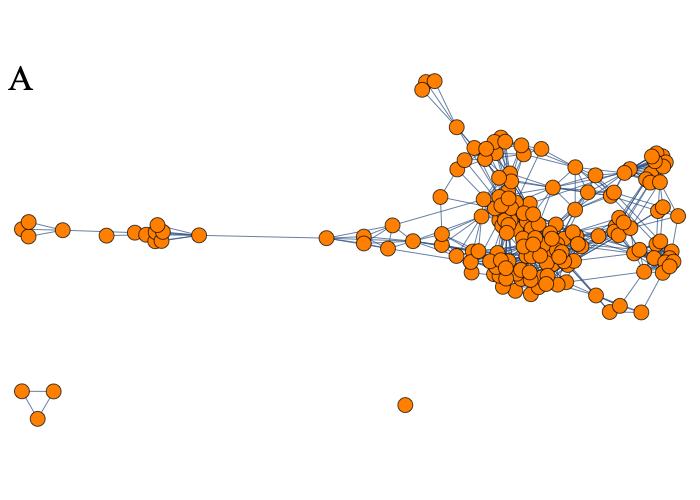 | 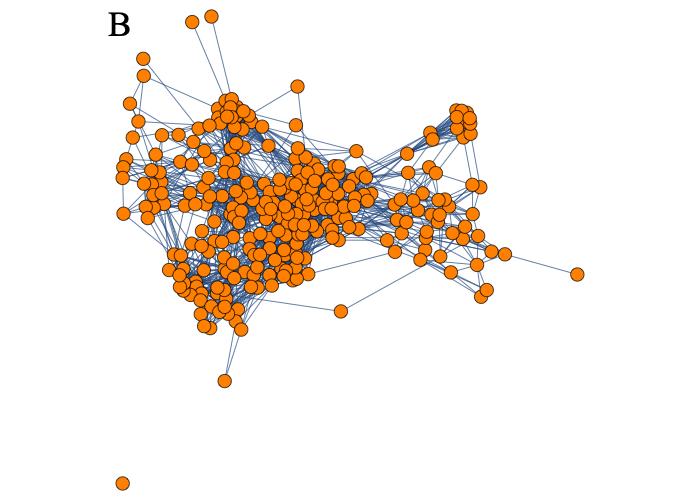 |
| --- | --- |

**Figure 2. H1N1 and HIV protein interaction networks.**Virus/protein-host/protein interaction network for A) H1N1 and B) HIV virus. We detect which protein produces a significant change in the average connectivity of these clusters by removing it from the network.

| 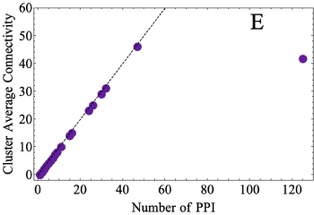 | 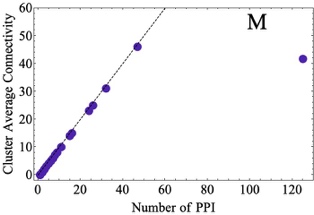 | 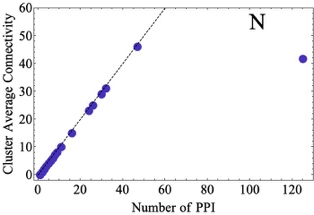 | 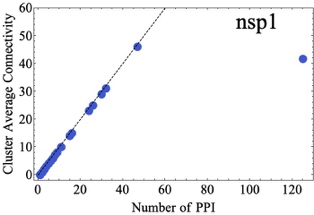 |
| --- | --- | --- | --- |
| 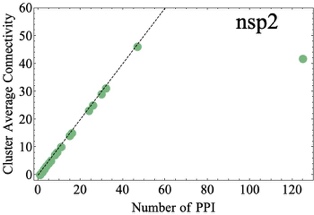 | 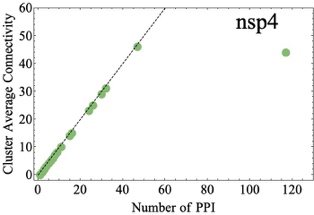 | 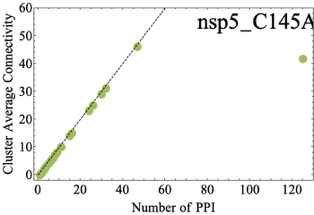 | 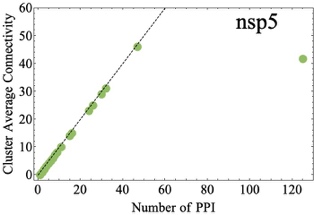 |
| 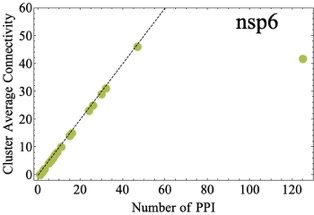 | 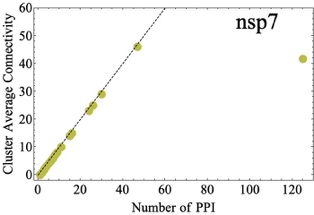 | 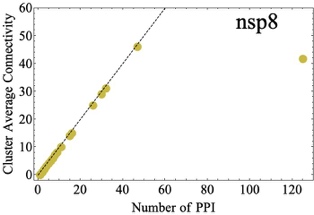 | 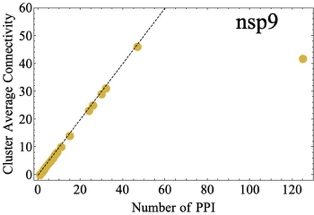 |
| 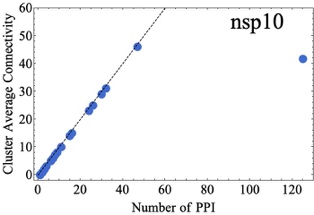 | 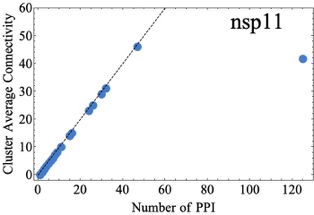 | 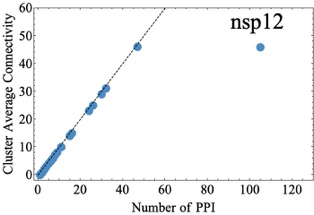 | 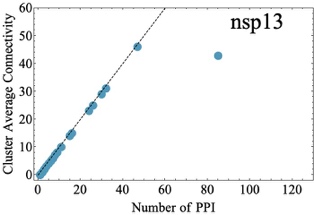 |
| 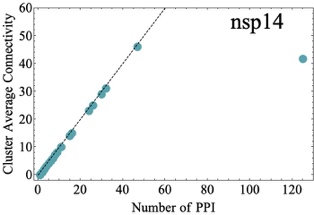 | 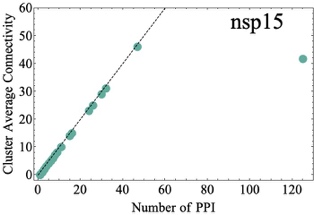 | 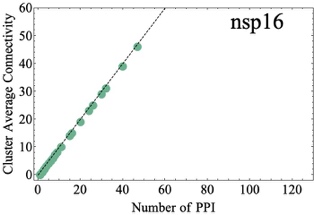 | 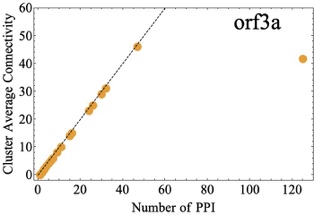 |
| 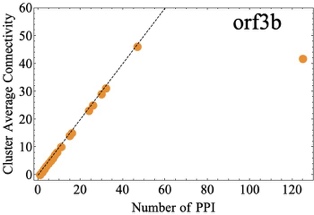 | 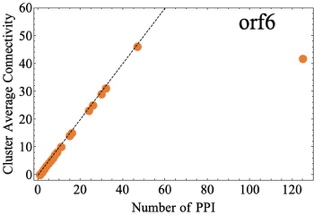 | 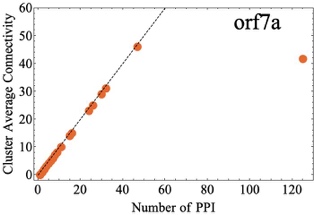 | 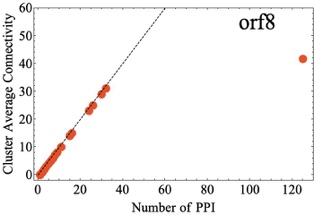 |
| 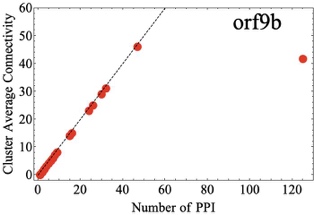 | 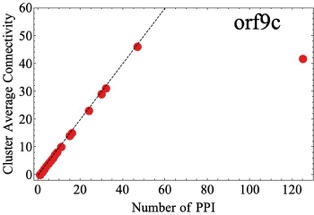 | 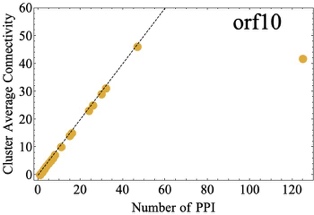 | 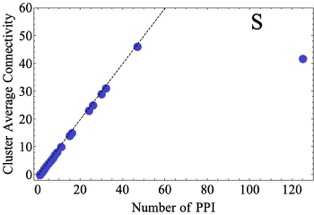 |

**Figure 3. Host-coronaviruses protein interaction network SARS-CoV-2.**Cluster average connectivity dependence with the number of protein interactions once a single virus protein has been removed. Color code emphasizes the effect of the removal of different proteins.

| 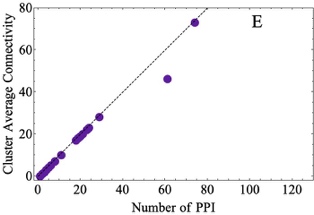 | 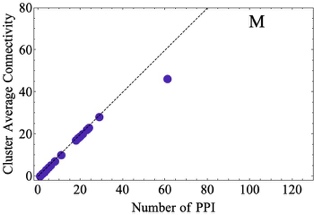 | 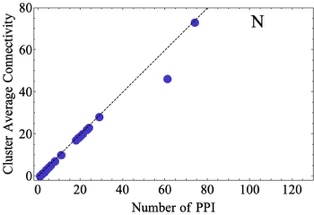 | 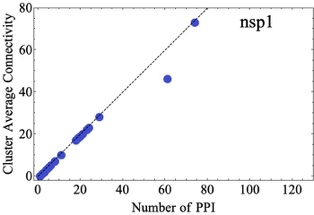 |
| --- | --- | --- | --- |
| 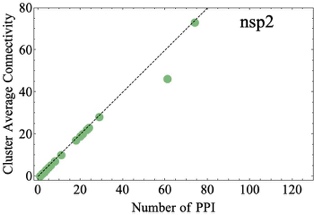 | 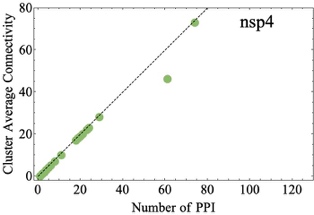 | 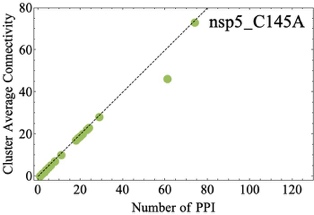 | 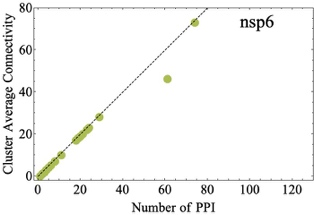 |
| 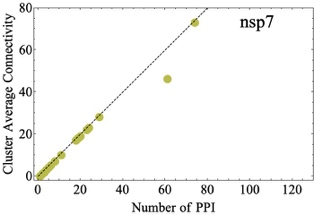 | 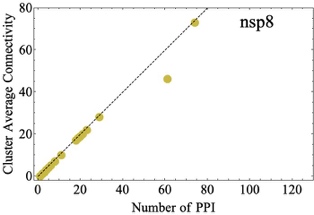 | 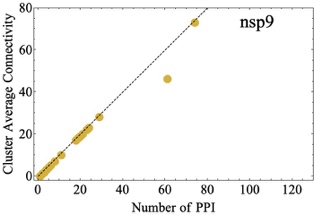 | 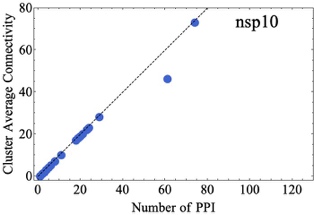 |
| 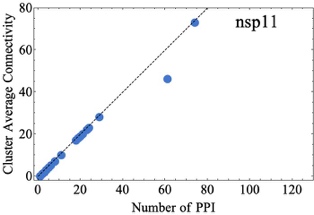 | 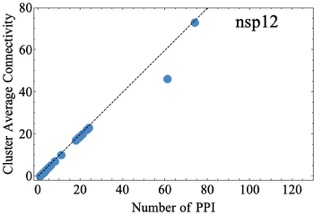 | 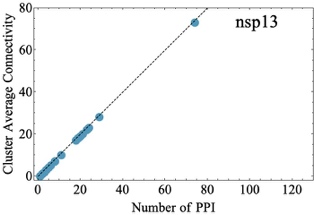 | 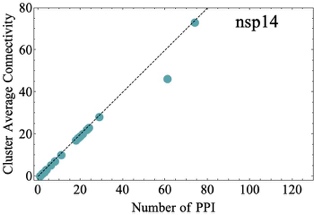 |
| 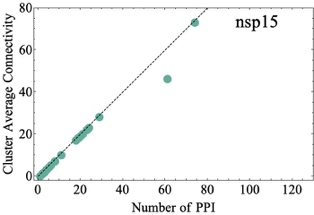 | 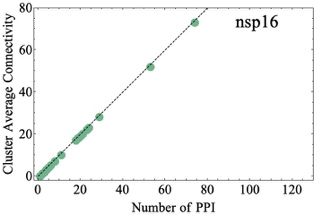 | 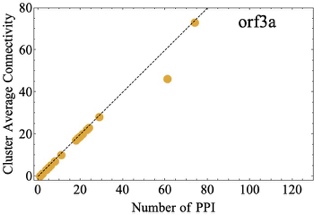 | 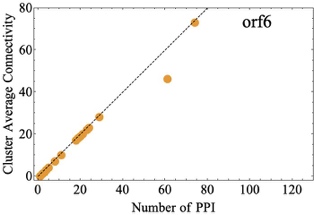 |
| 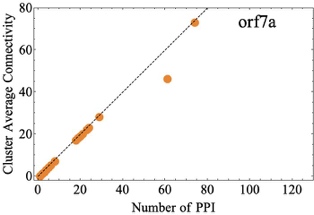 | 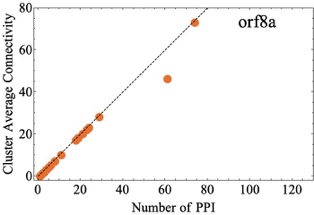 | 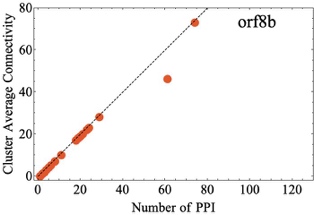 | 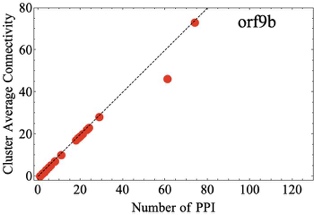 |
| 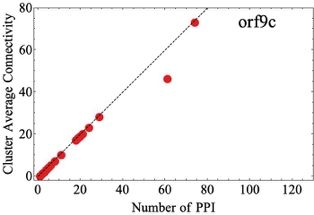 |  |  |  |

**Figure 4. Host-coronaviruses protein interaction network SARS-CoV-1.**Cluster average connectivity dependence with the number of protein interactions. once a single virus protein has been removed. Color code emphasizes the effect of the removal of different proteins.

| 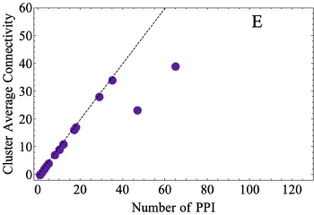 | 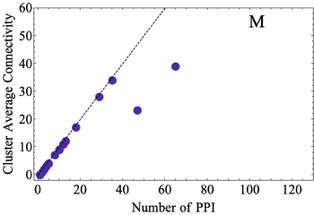 | 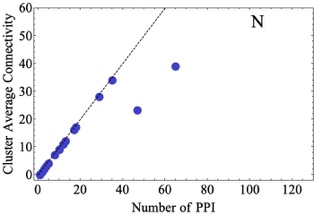 | 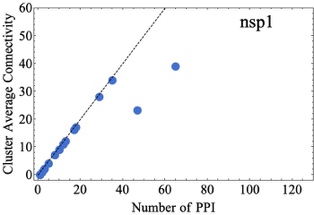 |
| --- | --- | --- | --- |
| 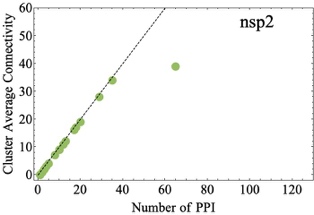 | 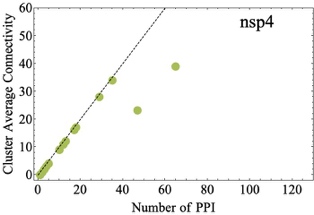 | 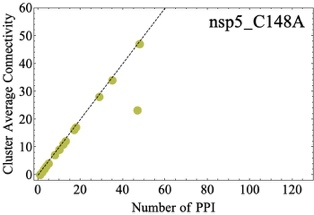 | 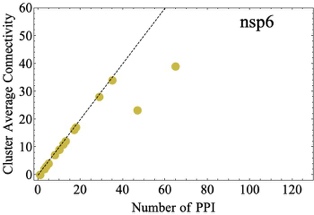 |
| 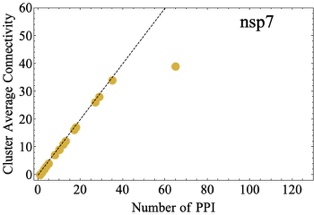 | 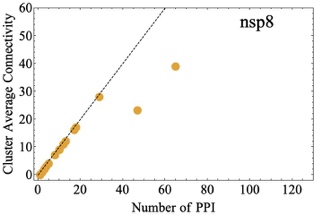 | 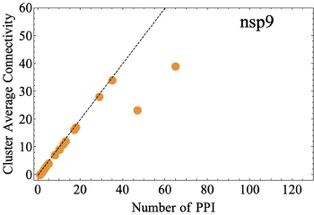 | 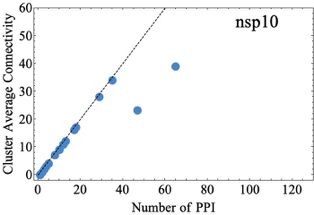 |
| 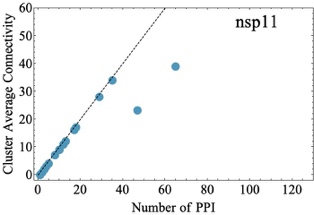 | 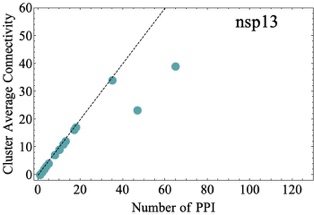 | 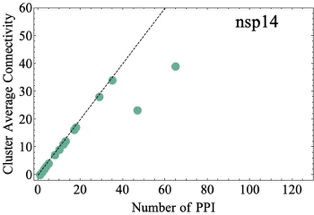 | 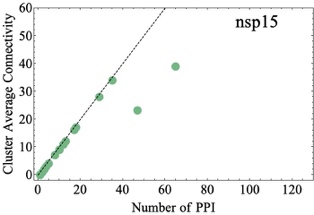 |
| 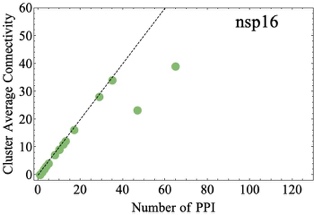 | 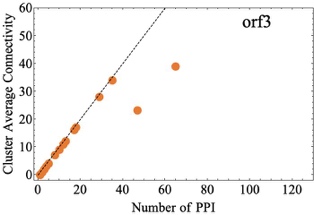 | 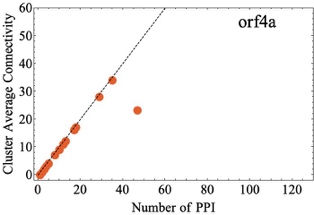 | 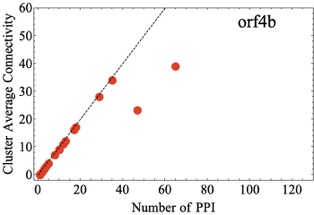 |
| 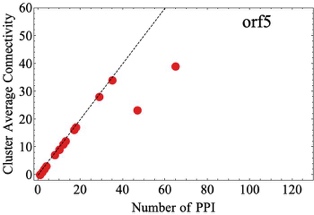 | 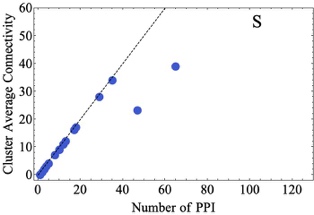 |  |  |
|  |  |  |  |

**Figure 5. Host-coronaviruses protein interaction network MERS-CoV.**Cluster average connectivity dependence with the number of protein interactions once a single virus protein has been removed. Color code emphasizes the effect of the removal of different proteins.


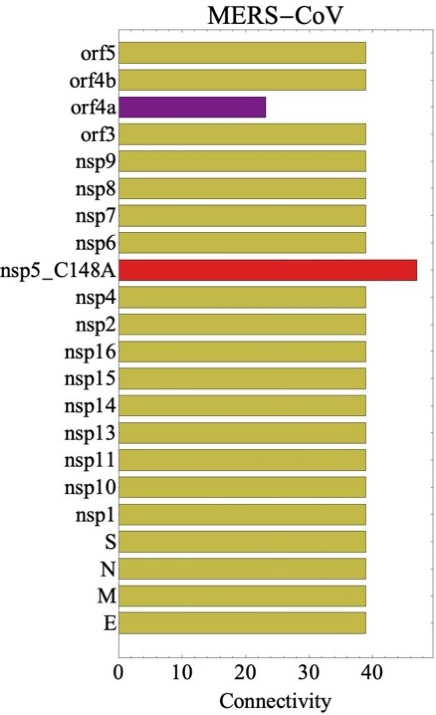

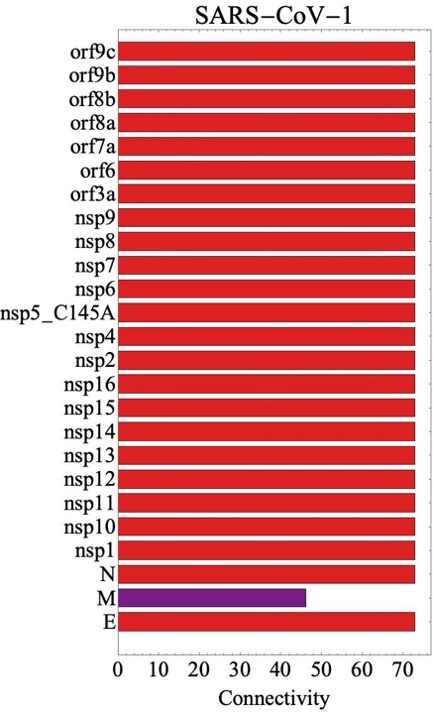

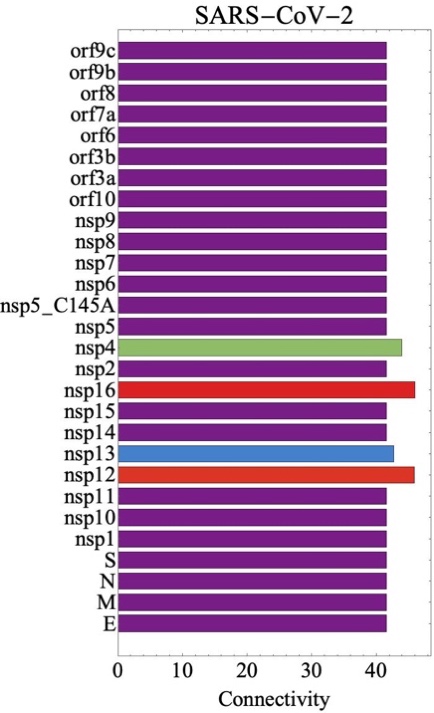


**Figure 6. (Color online) Largest cluster analysis of host-coronavirus protein networks.** Change in the average connectivity of the largest cluster upon removal of a particular virus protein. From left to right MERS-CoV, SARS-CoV-1, SARS-CoV-2. Color code is included as a guide to the eye.


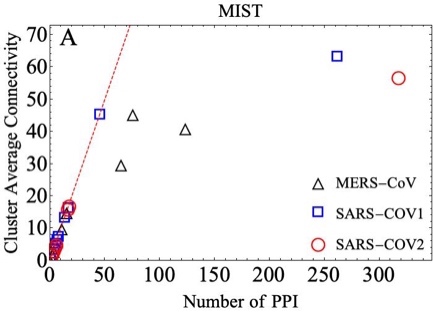
 **
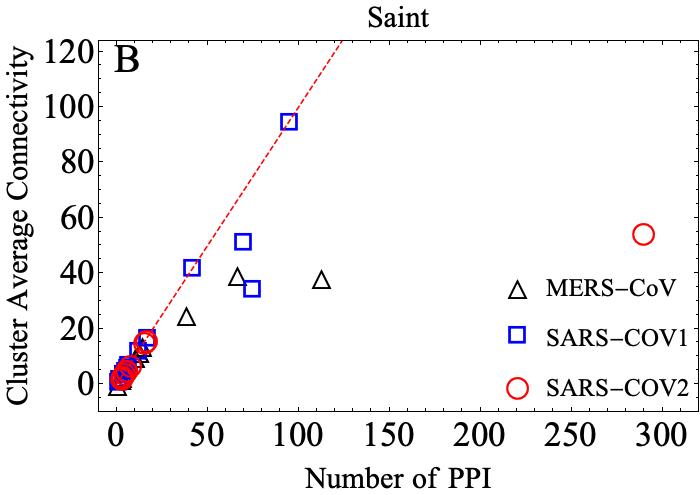

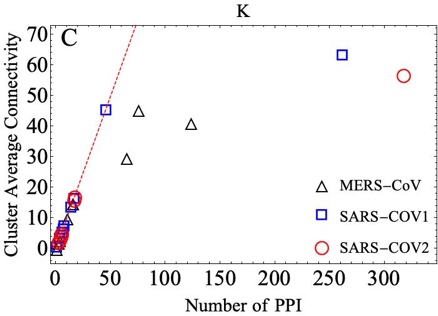
**

**Figure 7. DIS, Saint, and K-average protein networks.**Comparative analysis of average cluster connectivity dependence on the number of protein interactions for three statistical methods: MIST, Saint, and K scoring. These three cases display a linear relation for small clusters, as expected for a random network, while larger clusters deviate from these trends.

| 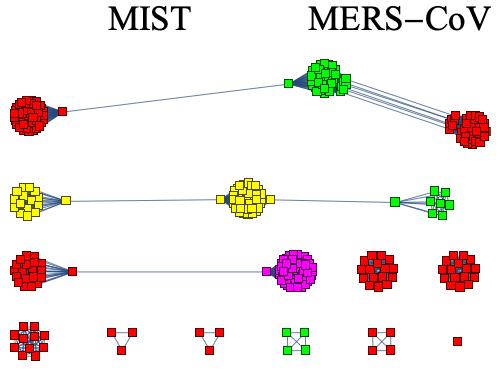 | 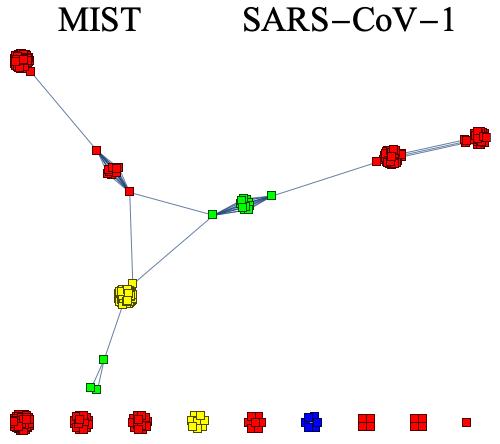 | 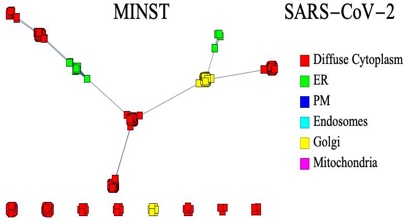 |
| --- | --- | --- |
| 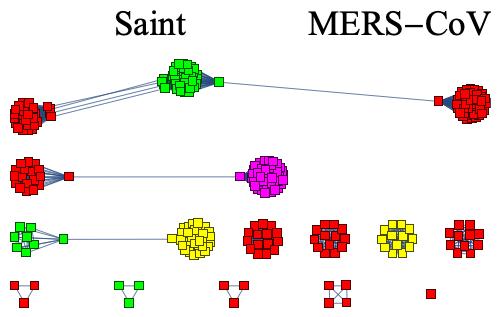 | 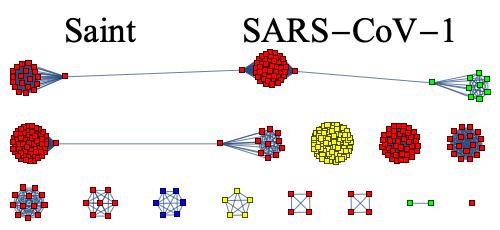 | 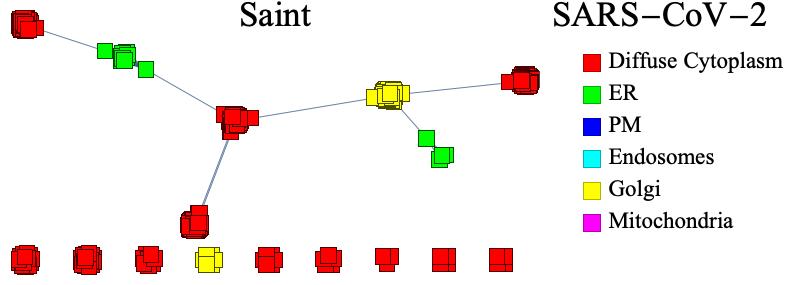 |
| 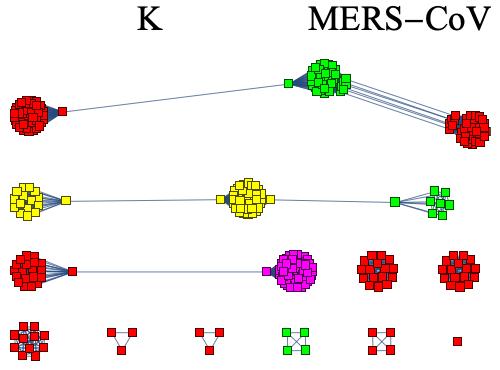 | 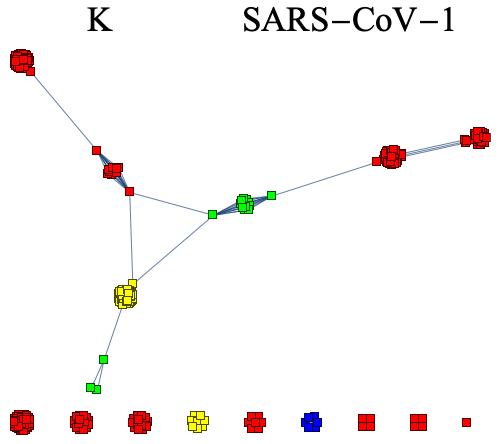 | 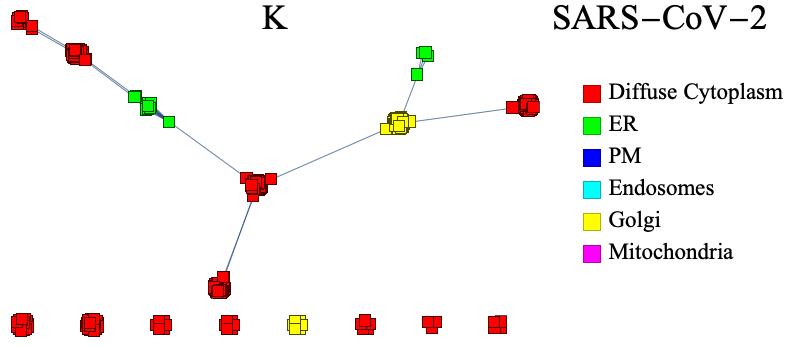 |

**Figure 8. Host-coronavirus protein interaction networks.**Comparative analysis of protein-interaction-based networks using different statistical scoring methods. SARS-CoV-2 and MERS-CoV preserve their cluster structure independent of statistical scoring method. However, the larger SARS-CoV-1 cluster is modified.


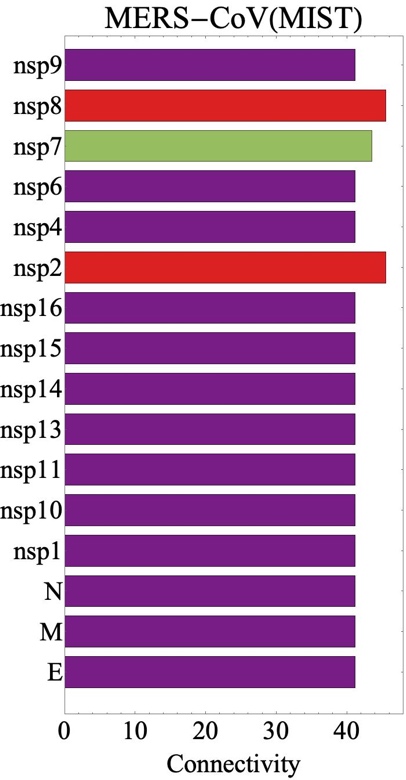

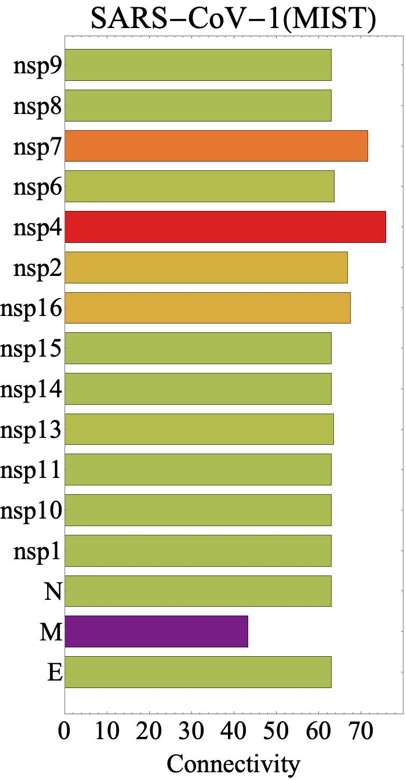

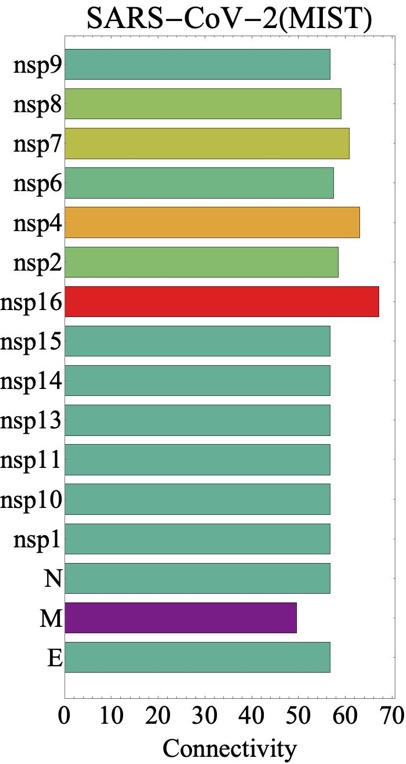


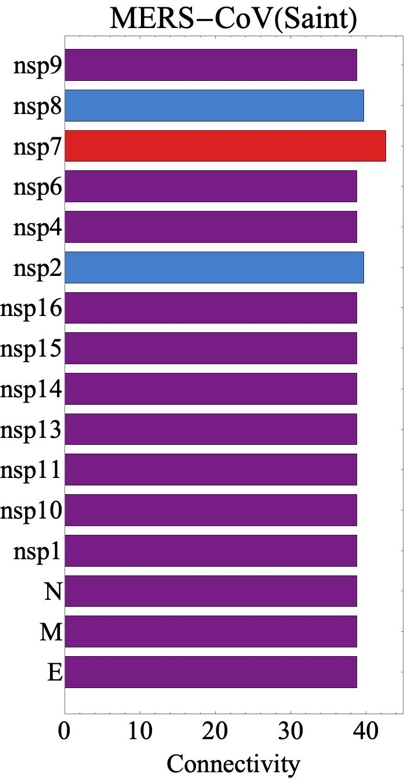

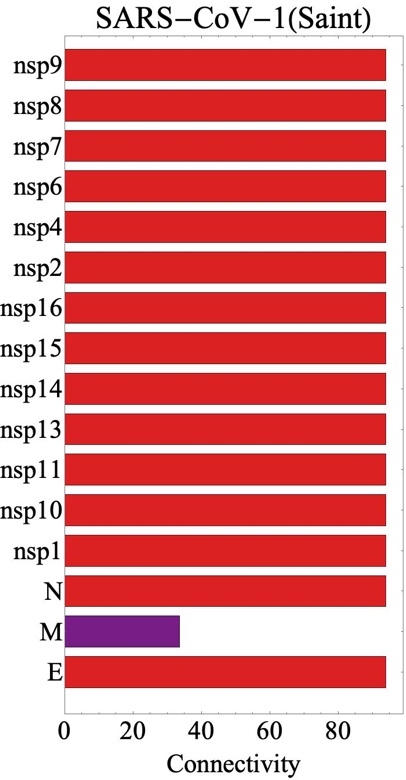

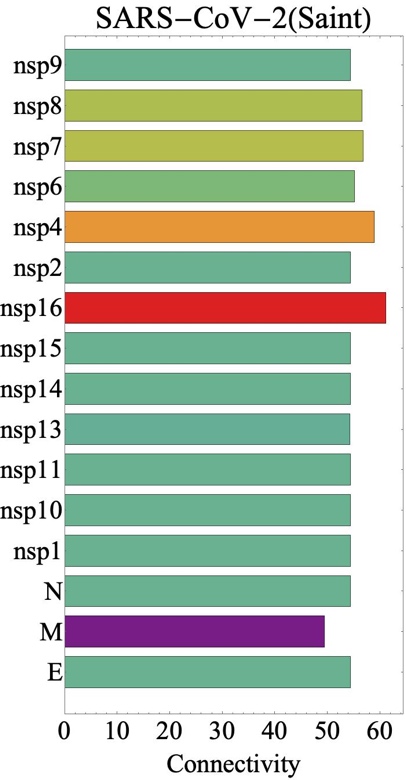


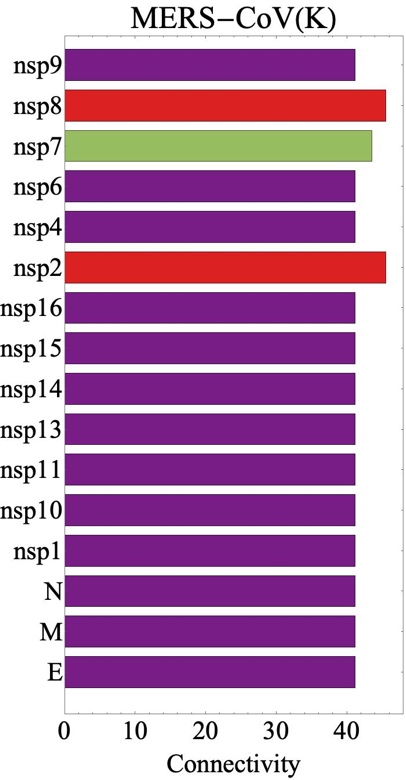


**Figure 9. Coronavirus Network's connectivity.** Change in the average connectivity of the largest cluster upon removal of a particular virus protein (color code is included as a guide to the eye). We analyze the MERS-CoV, and SARS-CoV-1, SARS-CoV-2 networks created using different statistical scoring methods.
